# Immunity for sale: depictions of immunity in British newspaper advertising, 1890–1940

**DOI:** 10.1017/mdh.2024.26

**Published:** 2025-01

**Authors:** Maebh Long

**Affiliations:** English Programme, The University of Waikato, Hamilton, New Zealand

**Keywords:** Immunity, Immunology, Medical advertising, Patent medicines, Preventative medicine, Resistance

## Abstract

This article analyses the depictions of immunity and immunological functions employed in proprietary medical advertising in British newspapers between 1890 and 1940. Using marketing copy to gain insights into the ways immunity was presented to the public and normalised outside of medical institutions and publications, I offer four main areas of discussion. First, I present an analysis of the ways advertisements evoked both natural and artificial immunity in their marketing copy, thereby affording us insights into the ways immunity was made palatable both to those supportive of and opposed to vaccinations. I then unpack the ways in which this advertising copy often emphasised immunity rather than the immunological, that is, presented immunity as resistance to infection achieved by purchasing particular brands, rather than as part of a defensive process taking place at a cellular level. Third, I examine the ways in which advertisements engaged with futurity and drew on a narrative of social exclusion that pitted created communities of the immune against the non-immune. Finally, I analyse the ways in which immunity was used to connect the biological and the psychological, looking particularly at the ways immunity against worry was sold to the public.

## Introduction

On 31 August 1899, an advertisement for the *Century Dictionary* gave a list of thirty words that ‘puzzle the general reader’.^1^ Included in the words ‘which the average man will hardly find it easy to define’ were ‘Antitoxin’, ‘Osteopathy’, ‘Aerobic’, and ‘Immune’.^2^ Yet a decade later the words ‘immune’ and ‘immunity’ were regularly used to market a wide range of patent medicines in Britain. Take, for example, the following playful advertisement from 1909 for Woods Great Peppermint Cure, a popular cough and cold remedy:

Some politicians prate about Protection,
And some folks fuss about Free Trade,
And others still own to a predilection,
That puts such abstract problems in the shade,
And say without the least circumlocution,
Mankind’s finest duty should be to secure,
In splendid health the human constitution,
Immune from colds by Woods Great Peppermint Cure.
^3^

Woods clearly considered the idea of being ‘immune from colds’ so readily understood by the public that it could be used in the final line of doggerel humorously blending debates about tariff reform and medicine. In the same year Phosferine, a tonic claiming to protect against ‘colds, chills, influenza, rheumatism […] neuralgia, muscular strain and vertigo’, announced that they had enabled immunity from disease in a broad cross-section of the British public, from steeplejacks to singers, cricket players to poets.^4^ Their advertisements’ use of the testimonial format, which gave the impression of quoted speech, shows that they considered ‘immunity’ to be a fitting word for local labourers and glamourous foreigners alike: in a campaign that ran for most of the 1920s, performers Fred Astaire and his sister Adele were cited as claiming that Phosferine’s ‘immunity from the usual nerve disorders, lassitude or exhaustion […] never lets us down’.^5^ By the late 1930s chemists felt comfortable including immunity in their New Year’s messages: ‘*The Directors of J.W. Simpson (Chemist) Ltd. wish you Immunity from disease, Perfect Health, Peace and Prosperity throughout the Coming Year*’.^6^ From 1890 to 1940 medical immunity grew from a term considered perplexing to the average reader and rarely drawn on in advertising to one wielded widely by the advertisers of patent medicines. It became a popular part of advertisers’ sales arsenal, used to evoke an often contradictory range of meanings and political positions, from natural immunity to acquired immunity, specific resistance to general resistance, medical protection to political exemption. As we see below, advertisers frequently drew on this breadth of signification to appeal to as many readers as possible.

**Figure 1.**
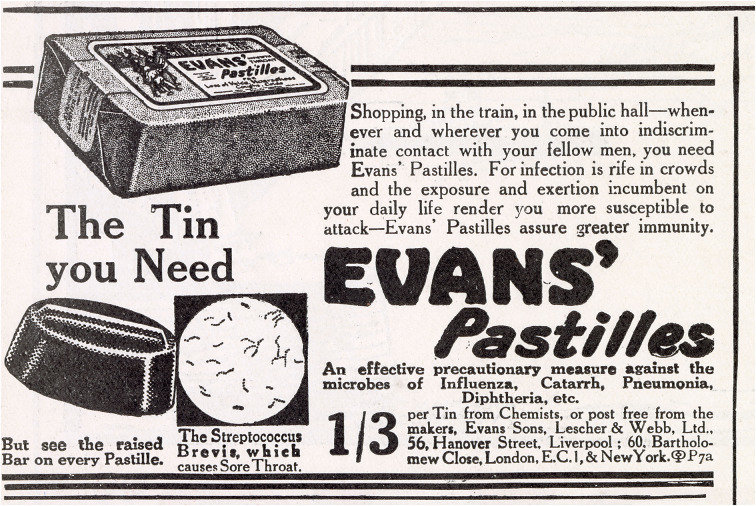
Evan’s Pastilles. Source: *The Sketch*, 27 October 1920, 67. © Illustrated London News Ltd/Mary Evans.

The late nineteenth and early twentieth centuries saw a huge growth in the numbers and kinds of proprietary medicines available for general purchase in Britain, as reasonably priced pills and tonics could be bought in pharmacies, grocers, department stores, barbers, tobacconists, and bookshops. Manufacturers advertised widely and regularly, frequently using hyperbolic language that blended the emotive and the scientific. Their use of technical terms was broad and increasingly incorporated the growing fields of immunology and bacteriology. The 1880s had heralded a period of rapid advancements in immune responses, and from 1890 immunity became a frame for evoking a life of exemption from illness in the marketing of remedies for colds, influenza, coughs, and nervous disorders (Figure 1). These were diseases for which there was no definite or ready cure, but which were frequent occurrences with a decided impact on quality of life. Within the context of a growing science of preventative medicine, which is a science of health as well as one of disease, immunity became and remains a powerful marketing force.

This article arises from a detailed study of every advertisement that used the terms immune or immunity in newspapers published between 1890 and 1940 and digitised in the *British Newspaper Archive.* It presents an analysis of four significant themes in the descriptions and affective discourses of immunity. These themes reveal the complexities and contradictions attendant on the birth of immunity as a powerful rhetorical concept of embodied resistance to threat. Unpacking the early decades of discourses of immunity, specifically those that take place outside of scientific journals, medical pamphlets, and doctors’ offices, shows the ways immunity was embedded in a nexus of capitalism, social hierarchies, and contemporary concerns regarding vaccinations, natural health, and mental wellness. Collectively the themes reveal the ways the use of immunity within a marketing context frequently decoupled it from immunological processes, presenting it instead as a condition or state achieved by the purchasing of patent medicines. Immunity, these advertisements imply, is a mode of perfect health inextricable from commodities and capitalism, as whether the advertising copy aligns itself with discourses of nature and natural immunity, or draws on the language of immunology and bacteriology, it insists that to be immune is to have gained a state of wellness by buying and consuming tonics and tablets. Immunity, these advertisements told readers, is less a cellular or embodied process than the end result of sensible shopping. Once immunity entered advertisers’ vocabulary as an evocative rhetoric of resistance it never left. That noted, this article uses 1940 as an end-point: the Second World War, the beginnings of the antibiotic era, and changes in immunology brought about by Frank Macfarlane Burnet mark a new stage in discourses of medical resistance and threat.

Following detail on the context within which a discourse of immunity grew, I outline advertisers’ handling of the developing concepts of natural and artificial immunity, focusing on their language use to map the ways they navigated the polarising issue of vaccinations. I then provide insights into the ways advertising copy frequently presented a skewed relation between immunity and immunology, as the desire to sell products meant that advertisers figured immunity as the prevention of disease achieved using their brands, rather than as part of a defensive process taking place at a cellular level. This section provides insights into the ways the public might have misunderstood the functioning of what we now call the immune system.^7^ It also explicates the ways immunity was embedded in external, purchasable products rather than bodily processes. The third section maps the temporal and social aspects of the advertisements, looking at the ways immunity was connected with futurity and the creation of a community of the immune, a rhetorical strategy bringing immunity into discourses of the body politic and anxieties about degeneration and social decline. The final section traces the ways immunity was used by advertisers to connect the health concerns of the body and the mind, as the discourse of immunity was used to sell the concept of a life free of worry. Although my analysis draws on the implications of advertisers’ misrepresentation of immunological and bacteriological concepts, my primary focus is on the insights into public knowledge we can glean from the way advertising copy wielded, adapted, and transformed immunological discourse, rather than a simple evaluation of the truth of the claims made by brands.

## Background

In H.G. Wells’s enormously successful novel, *The War of the Worlds* (1898), humanity is brought to the brink of defeat by the Martian attack. The Earth is saved only when the invading aliens are overcome by pathogens to which humans were ‘altogether immune’, but against which the invaders had no ‘resisting-power’.^8^ As *The War of the Worlds* exemplifies, the reading public in the late nineteenth century was growing accustomed to the invisible hazards posed by germs, in which ‘Every sneeze means danger of infection, as ninety-nine times out of a hundred someone catches the flying germs’.^9^ As lay audiences grew to understand the presence of the microbial, the modern world appeared rife with invisible threats, causing sceptical writers like G.B. Shaw to complain that it was a ‘marvel that anyone could possibly survive three days in an atmosphere consisting mainly of countless pathogenic germs’.^10^ But as bacteria galvanised public fears, immunology reassured the public of bodily defences, a reassurance that companies were quick to incorporate into their marketing. An advertisement for Hall’s Wine in 1910 is exemplary: ‘Let the winds blow, let the air be charged with microbes, let infection surround you as it will, you will be immune to all dangers if you have fortified yourself with Hall’s Wine’.^11^

Brands like Hall’s Wine and Lifebuoy soap could promise that they could make consumers ‘healthy – immune from the germ-laden things which scientists tell us you are bound to come in contact with daily’,^12^ because of the rapidly growing fields of bacteriology and immunology. Early advancements in disease prevention were brought about by Mary Wortley Montagu’s promotion of smallpox inoculation in England in the early eighteenth century and Edward Jenner’s use of cowpox to vaccinate against smallpox in the late eighteenth and early nineteenth centuries. From these forays into resistance Louis Pasteur’s work on acquired immunity and chicken cholera in the 1880s heralded a new age of immunological research, with Élie Metchnikoff publishing a cellular theory of immunology in 1884, Emil van Behring and Kitasato Shibasaburō releasing their findings on diphtheria antitoxin in 1890, and, in 1897, Paul Ehrlich proposing his side-chain theory of antibody formation.^13^ Seeking a name for the state of being spared sickness, the scientific community had used ‘immune’, borrowing a word that had originated in ancient Roman law as the legal idea of exemption from political service or public duty. In the Middle Ages, it described the exclusion of the church from state laws and gradually grew to mean exemption from onerous duties. Through scientists’ adoption of the term, political exemption became medical resistance, and this usage expanded from scientific journals to popular publications.

As Metchnikoff said in his Nobel lecture in 1908, ‘There is no need to be a doctor or a scientist to wonder why the human body is capable of resisting so many harmful agents in the course of everyday life’, nor to question why, for ‘some individuals who go down at the attack [of an illness], there are others who have immunity to a greater or lesser extent’.^14^ Metchnikoff and his fellow scientists provided the public with a way to understand and imagine bodily resistance, as well as the occasional failures of that resistance, to infection. When Wells brought defence and resistance together in a scene that resolves interplanetary conflict through bacteriology, he captured the compelling rhetorical force that immunity could attain. Immunity became the term for the shield or barrier that thwarted a not-yet-present illness and insured against future risk.^15^ Prevention attained a whole new importance, indeed, in *The Modern Family Doctor: A Guide to Perfect Health* (1914), it is Edward Jenner who is described as the father of preventative medicine.^16^

As immunity featured more regularly in advertising copy its range expanded to articulate an array of concerns regarding the boundaries and vulnerabilities of the body and body politic. This expansion was bound up in the rise of modern health culture, a large part of which was inextricable from the idea of the healthy body as a duty to the self, state, and Empire, but also as a purchasable commodity. Concepts of self-help and physical well-being enabled the public to have an increasing sense of agency regarding their health, and the growth in the advertising of patent medicines and cure-alls encouraged them to believe that self-diagnosis should be followed by self-prognosis and self-medication.^17^ It also enabled agency during a period in which medicine was increasingly technical, which could turn the patient into a passive participant in their treatment.

From the late nineteenth century, there was an increase in the range of illnesses for which doctors were normally called, and which they could cure. The sales of patent medicines, however, which patients could self-prescribe, also rose.^18^ Doctors were unable to cure colds and influenza or create a state of complete well-being for their patients, so the public added a range of therapeutic and preventative wellness activities to their arsenal of doctor’s prescriptions and domestic remedies. Trends such as the mind-healing movement of New Thought and Émile Coué’s *Self-Mastery Through Conscious Autosuggestion* (1922) were joined by vegetarian diets and ‘simple’ eating, rigorous exercise and fresh air, sun-bathing, staying thin, as well as by patent medicines that promised self-healing for everything from nervous maladies to influenza. Growing in tandem with self-help initiatives were depictions of the early twentieth century as over-medicalised and as an ‘age of worry’, with growing disquiet about the mind’s ability to endanger the body by allowing concerns to overwhelm it.^19^ For Andrew Wilson, a Scottish zoologist writing in 1900, the pace of modern life meant that the early twentieth century was a period of ‘little health’. ‘We are’, he wrote, ‘not exactly ill, and we are not precisely well’.^20^ While certain diseases were being controlled, Wilson considered worries and nervous conditions to be on the rise. There is, he stresses, ‘no panacea for the modern disease of nervousness, and for the irritabilities it carries in its train’, and yet fortunes were being made by the sellers of patent medicines, as the modern age had become ‘a pill-swallowing age, a potion-loving generation’.^21^

There were numerous nerve tonics and pills on the market, including those like Sanatogen, Wincarnis, and Phosferine that promised immunity to neurasthenia and anxiety, and popular texts of the time brought discourses of immunity into conversation with questions of mental distress. In *Worry: The Disease of the Age* (1907), C.W. Saleeby, a eugenicist until the 1920s, argued that worry and negative attitudes play a vital role in determining an individual’s susceptibility to illness. In a chapter on ‘worry and disease’, he engages not only with worry but with the ‘whole problem of immunity’. Describing it as ‘perhaps the most complicated and obscure in the whole field of the medical sciences’, he argues that bacteriologists have not taken sufficient notice of ‘the importance of the mind in relation to infectious disease’.^22^ For him immunity is subject to worry, or, as British surgeon Haydn Brown’s pithy formula in *Worry, and How to Avoid It* (1900) put it, ‘worry causes illness, and illness causes worry’.^23^ As the title of Brown’s volume implies, the individual could counter worry by thinking differently about qualms, or, significantly, by applying a slight worry as a ‘counter-irritant’. Likening this process to the mind vaccinating itself against anxiety, Brown concludes his argument by noting that the

great Jenner found that in some instances a slight form of disease prevented a more serious one, and he instituted vaccination for people who wished to avoid small-pox. Worry should be dealt with in much the same manner; an attenuated mental exercise should be induced, so that the troubled brain may be brought under its antidotal and beneficent influence.^24^

For Brown, engagingly quickly with small worries would vaccinate the mind against larger worries that could overwhelm its defences. We see a similar connection between a rhetoric of immunity and mental health, if from a different ideological position, in a lecture given in 1937 by Maurice Beddow Bayly, an anti-vivisectionist and anti-vaccinator. In this talk Beddow Bayly promoted natural immunity over artificial immunity, arguing that the public’s ‘minds must become immune to fear’ before they could be healthy, and only by achieving greater understanding, compassion and selflessness could one achieve ‘a Natural Immunity to any disharmony or disease which might assail his Citadel of Peace’.^25^

Companies such as Beechams sold to ‘an ever-growing number of people who yearned for good health but kept clear of doctors’, as well as those who sought to supplement the treatment their doctors could provide. As a result, sales of proprietary medicines in Britain were about four million pounds at the end of the nineteenth century and rose to five million in 1914.^26^ In 1886 the *Chemist and Druggist*, a trade journal, explained the ‘unquenchable demand of the public for put-up medicines’ as follows:

The majority of people get to feel very bad before they go through the processes of being prescribed for and dispensed for in the orthodox fashion [by registered doctors]. But something definite, tested by experience, something they can purchase at a fixed price and by just naming the article, without being chastised as to all their physical miseries, will always be popular.^27^

This was echoed in 1910 by the *British Medical Journal*’s report on patent medicines, which speculated, with some irritation, that ‘[t]here are probably few, if any, ailments more frequently treated by the sufferer or his friends, without recourse to medical advice, than coughs and colds’.^28^

Manufacturers of patent medicine traded on the public’s desire for a self-directed treatment, which they afforded a quasi-medical status by legitimating their products, and the narrative around their products, through scientific language. Despite the prominence of medical language and testimonies by doctors in their advertisements, on the whole, as Takahiro Ueyama has argued, patent medicine manufacturers were less interested in the seriously ill than ‘with the minds and imagination of consumers worried about their health’. This fact, he notes wryly, is unsurprising, ‘given that the market potential for people concerned about their health far exceeded the market potential for people already in bad health’.^29^

Part of the power of immunity as a marketing tool was its ability to evoke diverse approaches to health. The affective and associative range of immunity held that, on the one side, immunity was antitoxins, immunisation programmes, and the power to conquer smallpox. On the other, it was innate vitality, natural strength, and pure blood. In signifying both active artificial immunity, which tended to suggest the realm of doctor–scientist, as well as natural immunity, more readily the space of the patient–consumer, immune discourses in marketing evoked protection and resistance across ideological divides. More specifically, immunity’s polysemy meant that it could navigate the fault lines between those opposed to vaccination and those who saw its importance, an important attribute for a period in which conscientious objectors were still resisting vaccinations. Periodicals such as *The Vaccination Inquirer and Health Review* (1879**–**1958) and *The Abolitionist* (1899**–**1948) and works such as Alexander Paul’s *The Vaccination Problem in 1903 and The Impracticability of Compulsion* (1903) frequently criticised vaccinations as introducing impurities into the bloodstream, were often strongly opposed to vaccinations’ connections with vivisection, and argued instead for greater attention to sanitation and public health.^30^ For those suspicious of vaccinations, then, the products sold through the promise of immunity could be presented as boosting the consumer’s natural immunity.

Similarly, while ‘immunity’ is a specific and technical term, the idea of resistance could be, and was, used loosely, expansively and imaginatively. This is particularly the case as the products were primarily sold as treatments and preventatives for coughs, colds, and influenzas and extremely rarely for notifiable illnesses. We do, however, need to be cautious about an easy dismissal of the kinds of infections for which brands promised immunity. The 1899–1990 outbreak of influenza, often referred to as Russian Influenza, was the worst pandemic of the nineteenth century and is estimated to have caused one million deaths worldwide.^31^ There were other bad influenza epidemics in the early twentieth century, before the devastation of 1918**–**19. Nor was influenza an easy disease to treat: Lori Loeb argues that orthodox therapeutic practice was often very similar to the ‘quackery’ of patent medicines and cure-alls that doctors regularly dismissed.^32^ Treatment usually depended on rest and good nutrition, with alcohol prescribed in often large doses. The dismissive ingredient breakdowns that featured in reports on the composition of secret remedies belied the fact that while some remedies did contain harmful ingredients such as lead, others were merely harmless, and some beneficial, as they included content such as iron, bicarbonate of soda (such as Eno’s Fruit Salts), and rhubarb as a laxative.^33^ As such, we cannot assume that consumers purchasing the products advertised in newspapers and magazines were making inappropriate choices – while advertising overpromised and was designed to sway, consumers were still attempting to make informed selections and were often following what was considered best practice at the time.

The careful deliberation of consumers was, however, pitched against the tendency of modern advertising to sell a better life rather than a single product. As Raymond Williams wrote in 1961, it is ‘impossible to look at modern advertising without realizing that the material object being sold is never enough: this indeed is the crucial cultural quality of its modern forms’.^34^ We have, he stresses, a system in which the objects being sold must be validated by larger significance, that is, by ‘*magic:* a highly organized and professional system of magical inducements and satisfactions’, not only similar to magical or totemic systems but ‘rather strangely coexistent with a highly developed scientific technology’.^35^ The magic of advertising obscures the difference between an individual as a consumer and as a user: the public is encouraged to shop aspirationally, purchasing the fantasy that a product promises rather than to shop based on what the product actually does. In the instances analysed in this article, immunity helps to imbue commodities with their magical, talismanic quality. Its power comes from its combination of magic and scientific control: the power of immunity, a modern silver bullet, is promised against the risks of the world, be they germs, spoiled food, or drops in temperature. Modern science is tied to a modern mastery over the world’s dangers, many of them invisible, through the ‘magical’ qualities of immunity. Of course, the magical thinking bound up in advertisers’ discourses of immunity means that they often convey blurry pictures of immunological processes – this is particularly clear in the section below on immunity as a prize rather than an immunological process – and frequently cross into the space of exaggeration and broad promises commonly depicted as quackery. The implications of the magical thinking around marketing with immunity are outlined in the following four sections.

## Natural and artificial immunity

The most commonly evoked magical quality of immunity in advertising was that of a perfect, unassailable shield. In this and the following sections I unpack significant and nuanced representations of immunity, but these instances of subtlety are built on the fundamental understanding of immunity as impregnable resistance. Vin-Si-Co, a tonic wine, is a typical example. ‘Make yourself immune’, the copy urged readers, ‘from the dreaded scourge of Influenza! Fortify your entire system so that the insidious ‘flu germs are powerless to penetrate your natural defences’.^36^ Immunity, the advertisement claims, is a fortification that cannot be penetrated, although the copy insists that ‘natural defences’, that is, the body’s immune system, require the boost given by tonic wine to operate fully. The picture of strong fortification and enveloping defences gained further potency from rhetorical devices like the metonym and synecdoche. That is, in selling products that claimed to make bodies immune to the cold, cuts immune to infection, or stomachs immune to upset, advertisers sold the idea of being generally, existentially immune. The immune part conjured up the immune whole, and immunity’s mitigation of a specific threat to an aspect of life evoked a life of absolute protection and well-being. In 1932 Kee-pon throat sweets, for example, used a tagline that read: ‘*Keep out germs*! “Kee-pons” will keep you immune’.^37^ While the kinds of germs might be implied, the advertisement’s lack of specificity around the threat is matched by the nature of protection promised: a general state of immunity. The greatest modern vaccines, these advertisements suggest, come as pills and tonics that immunise the consumer against all forms of ill health and discomfort. This lure is what Jackson Lears famously called the ‘therapeutic’ aspect of early twentieth-century marketing: ‘the promise that the product would contribute to the buyer’s physical, psychic, or social well-being; the threat that his well-being would be undermined if he failed to buy it’.^38^ Immunity becomes the *feeling* of being immune, which Mark Davis refers to as ‘affective immunity’: feeling protected and secure against threats visible and invisible, regardless of the actual efficacy of the products.^39^

The feeling of being immune, as the advertisements presented it, took two main forms: immunity imbued with the might of the natural and immunity fortified by the power of the vaccine. For those opposed to vaccinations, the immunity that advertisements offered was presented in terms such as those employed by Chymol tonic, which promised to ‘Build up your *natural resistance* to the all-pervading germs’ to ‘ensure your immunity from influenza or any other contagious disease’.^40^ Ovaltine reassured readers that ‘*you need not* be afraid of falling a victim to this prevalent [influenza] epidemic’, as if you ‘Keep up your bodily strength, increase your vitality’ with Ovaltine your ‘*natural powers of resistance* will be amply sufficient to keep you immune from danger’.^41^ Matea tried to blend nature and science by claiming its product had ‘*rendered the entire S. American continent immune from rheumatism*’ by being ‘*scientifically nature’s purest, most beneficial stimulant*’.^42^

To sell to those convinced of the importance of vaccinations, products became imbued with inoculation’s power. During the First World War, for example, Urillac tablets claimed to infuse ‘your whole circulation with an antidote to Uric Acid troubles, just as the Royal Army Medical inoculation renders British Soldiers immune from military epidemics even on the festering battlefield’.^43^ Describing rheumatism and gout treatment in terms of vaccinations, and evoking the conditions in the trenches to underscore both the pain caused by uric acid and the challenging conditions under which Urillac could perform, the immunity the tablets promised was supercharged by the power of inoculation as well as by the defensive systems of war. It was along similar lines that NDK Iodised Dental Colloid presented their toothpaste as a vaccine: ‘You bid Good-bye to Tooth and Mouth Troubles because Your immunized gums, *teeth* and mouth *defy* such things as Pyorrhea, Bleeding Gums, Gingivitis, and Unpleasant Breath’.^44^ Both categories of uses of immunity – those that drew on concepts of the natural and those that drew on associations with vaccinations – give us insights into the ways in which anti-vaccination ideologies were accommodated in mainstream marketing during this period, as well as the ways in which the technical language of the medical institution could be borrowed by products connected with alternative practices.

The varied positions immunity could be aligned with are encapsulated in an advertisement for Veno’s Seaweed Tonic from 1902. The copy, which has the wordiness of late nineteenth and early twentieth-century advertising, explains that when the ‘four great vital organs of the body’ – the stomach, liver, kidneys, and blood – are ‘sound and in a vigorous condition man is then in all his *natural glory*, an ideal specimen of humanity, free from ache or pain, moving about in the *full enjoyment of nature: immune from contagion or microbe*, a stranger to disease of any kind’.^45^ Readers of this advertisement could, depending on their preferences, read the mentions of immunity within the advertisement as part of a rhetoric of natural health or, guided by the advertisement’s germ theory references to contagion and microbe, position it within a medical/scientific discourse of acquired immunity. While the tonic is purportedly designed to boost the body’s resistance to infection, that is, to boost its natural immunity, the positioning of immunity after the colon, in the clause containing the language of bacteriology, connects it with modern investigations into acquired immunity. Furthermore, the colon draws a boundary – a physical representation of immune borders in the punctuation itself – between health, which is depicted as natural, and illness, which is depicted as unnatural, with the latter, the risk to well-being, marked by the language of medicine and science. Immunity is positioned after the colon, which places it in the realms of medical threat, but as the function of a colon is to indicate a description, definition, or explanation, then ‘immune from contagion’ becomes the definition of ‘the full enjoyment of nature’. With a clever sleight of hand by the copywriters, in this advertisement immunity is natural and acquired, a scientific term that is nonetheless part of the pastoral, a medical shield enabling the consumer to bask in the full enjoyment of nature. Depending on the consumer’s preferences, either the natural or the acquired aspects of immunity could be ignored.

**Figure 2.**
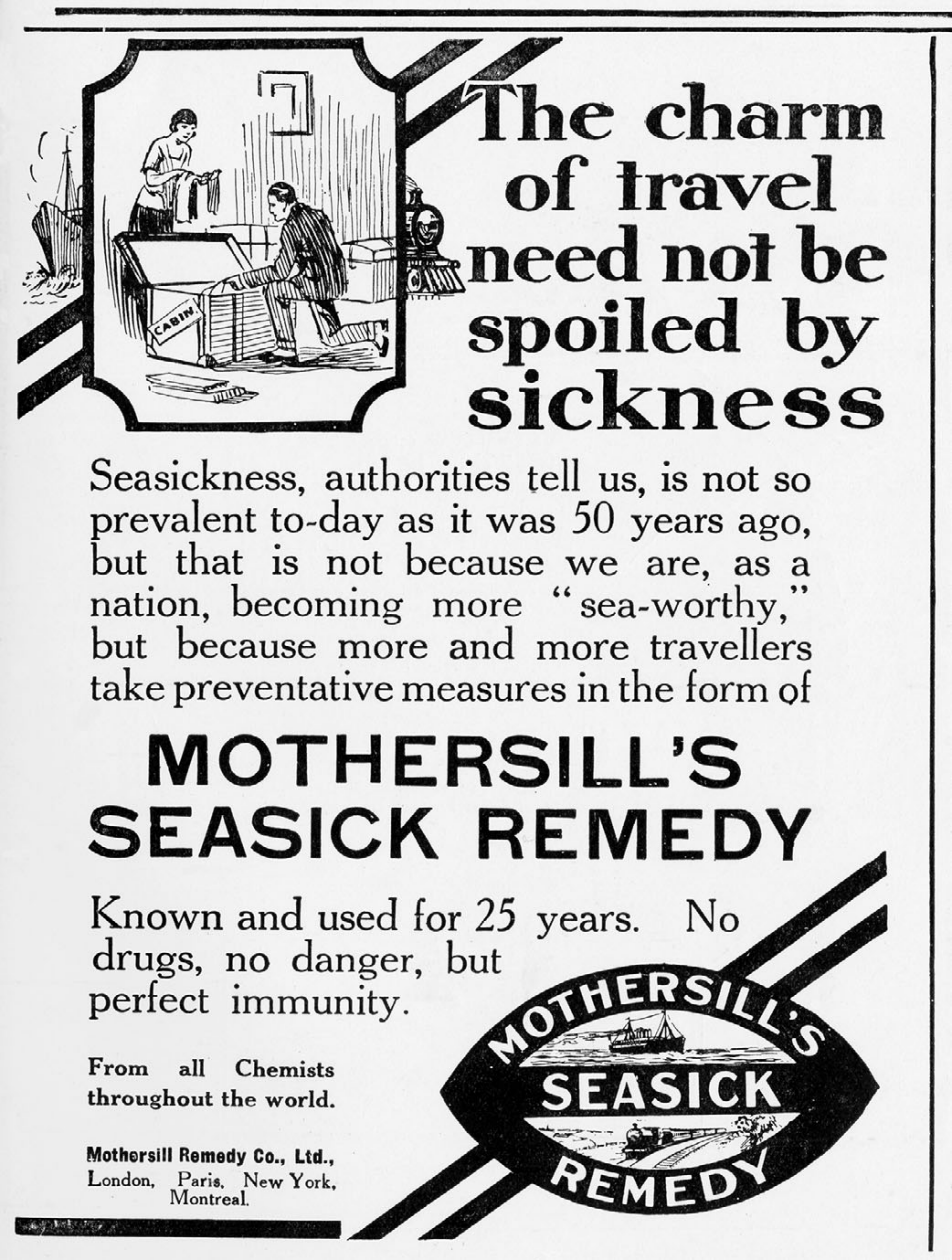
Mothersill’s Seasick Remedy. Source: *The Tatler*, 24 February 1926, 67. © Illustrated London News Ltd/Mary Evans.

Of course, given that the products in these advertisements were self-prescribed treatments that claimed to increase health and bodily resistance, that is, to increase natural immunity, it is unsurprising that discourses of nature are particularly prolific. Products were regularly marketed as natural remedies that provided immunity to the threats of unnatural modern life: irregular habits, fatigue, sedentary jobs, and urban living, which lead to an enervated, ill body. Tonics such as Wincarnis, ‘nature’s own remedy’, were pitted against the ‘attack of influenza germs’ through the claim that only ‘by these natural means can immunity be obtained from the dangers which are too prevalent in strenuous business life’.^46^ Sanatogen, ‘*the nerve tonic food*, supported its claim that ‘*Real immunity from illness is achieved only by keeping the nerves and blood nourished and fit*’^47^ with romanticised images of young men and women in togas in bucolic scenes. Similarly, Yadil antiseptic promised ‘Immunity from disease, boundless joy in living day in and day out, year in and year out’,^48^ while rejecting any association with modern medicine by juxtaposing their claims against images of youths in Greco–Roman tunics paying homage to the fountain of health, personified as a young woman in a waterfall. Patent medicines repeatedly presented themselves as part of ‘Modern medical science’, happily turning the mantra ‘prevention is better than cure’ into a pitch to sell medicine to the relatively healthy, but they did so in ways calculated to be palatable and unthreatening even to those opposed to immunity by injection. As Mothersill’s Seasick Remedy (Figure 2) assured readers: ‘No drugs, no danger, but perfect immunity’.^49^

**Figure 3.**
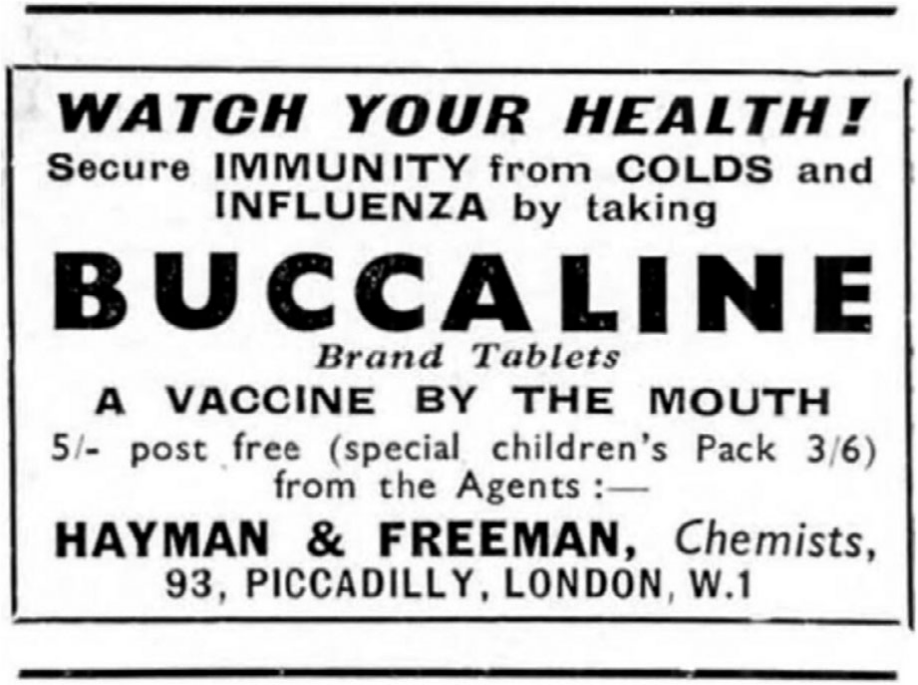
Buccaline Tablets. Source: *Country Life*, 30 March 1940, 2. © Future Publishing/Country Life.

In the 1930s, advertisements traded even more overtly on vaccination’s power to rapidly confer immunity while presenting their products as cleaner, easier, and less intrusive than an injection. ‘Protection by inoculation is doubtful’, the copy for Quincasca Cold Cure Tablets insisted, but their remedy was ‘most successful in conferring immunity from Colds, Catarrh and Headache’.^50^ Advertisers were potentially inspired by rhetoric such as that used by anti-vaccinators like Walter Hadwen, president of the British Union for the Abolition of Vivisection and vocal denier of germ theory. In 1921 Hadwen claimed that the moment the skin is broken by the needle ‘and noxious matter is poured into the underlying tissues, one of the strictest laws of Nature is outraged. […] Nature has provided her own path for the introduction of food or drugs into the system’, namely the mouth, ‘the only natural door of entrance’.^51^ This ‘natural door’ had clearly resonated outside of anti-vaccinators’ periodicals, with advertisements for Buccaline Tablets being a particular case in point: ‘*Vaccine by the mouth.* Get your Buccaline Brand Tablets now and secure immunity from colds and influenza for 4 to 6 months’.^52^ (See Figure 3 for an iteration with better image quality). Similarly, Anti-Bi-San tablets ran advertisements that promised ‘*Influenza Immunity without Hypodermic Injection*’^53^ as ‘this *dry vaccine treatment*, taken by mouth in tablet form, ensures absolute immunity. No injections – and no harmful reactions’.^54^ This was, the advertisers assured readers, complete immunity, with all the power of the vaccine, but none of the pain nor the vaccination association of needles.

## Immunity: a prize rather than a process

The use of militaristic language to describe germs and immunity is longstanding, and advertisements were no exception to this trend.^55^ The copy for Dr Slater’s Blood-Making Tablets, for example, which ensured ‘immunity from disease’, described influenza as ‘an enemy waiting outside the wall of bodily vigour […], and as colds, loss of tone, or any little ailment makes a breach in the wall, it rushes in and subjects the whole body to its ravages’.^56^ Congreve’s Elixir ‘*renders the subject immune against the attacks of the bacilli of consumption*’^57^ and during the First World War Urillac described itself as a ‘“Notice to Quit” to your health enemy’.^58^ The dangers of military rhetoric in health contexts are well-established^59^; instead, in this section I examine military language in patent medicine marketing to illuminate their emphasis on immunity, as a state of exemption that the healthy, protected body achieves, over the immunological, as the means through which the body resists infection. That is, the medical texts of the period used military language to describe immunological or bacteriological *processes*: Elizabeth Fraser’s *Manual of Immunity* (1912), for example, used phrases such as ‘the defensive armoury of the animal body’ and ‘big guns which are brought forth for the destruction’.^60^ Advertisements, however, tended to present immunity as the *result* of a struggle won by certain pills or particular tonics. Products like Bovril or Kruschen Salts were represented as the active combatants in the war against disease, with immunity as the prize won by their battle. To be immune, these advertisements implied, is to have created a wall or a shield or an impregnable defensive system, with their products its building blocks, as they provided the body with the conditions it needed to repel threats to its health. It is not that this presentation is wholly inaccurate, but rather that it is misleading, as it replaces the immunological processes that lead to immunity with a product that renders the consumer immune. As such, these advertisements give us important insights into the goal- rather than process-oriented ways the public might have envisioned immunity and immunological functions, and therefore the way immunity was embedded in the product rather than the person.

**Figure 4.**
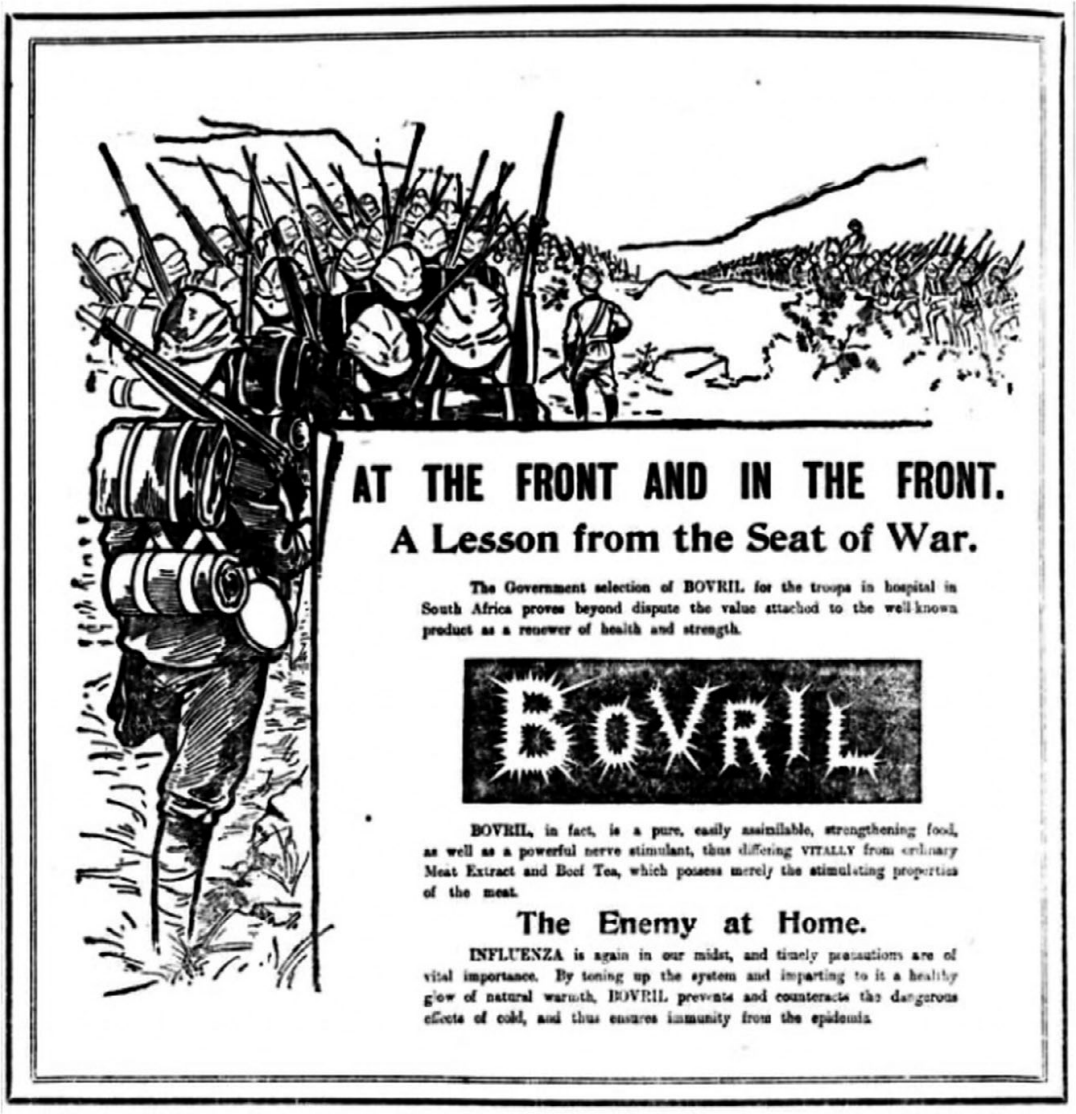
Bovril. Source: *Evening News*, 14 December 1899, 4. Content provided by THE BRITISH LIBRARY BOARD. ALL RIGHTS RESERVED.

Take, for example, Bovril advertisements during the Boer War (1899–1902), a conflict that has been repeatedly linked to British concerns about degeneration and poor health.^61^ Bovril, a liquid beef extract, was marketed strongly and successfully: its profits jumped from £18,977 in 1885, its first year in England, to £180,600 by 1900.^62^ During the winter of 1899–1900, Bovril advertisements regularly blended allusions to war with taglines about ‘*the enemy at home*’, stressing that ‘timely precautions are of vital importance’ if Bovril is to ensure ‘immunity from the epidemic’.^63^ This text (Figure 4) was repeated with different images across a wide range of newspapers, with its iteration in London’s *Evening News* featuring an evocative image of soldiers marching towards the front.^64^ This version informed readers that the British government had selected Bovril as part of the soldiers’ diet, which positioned Bovril ‘*at the front and in the front*’: the front line of (medical) defence for the soldiers on the front line. Bovril is presented as integral in the fight against germs and the Boer Republics, such that readers are told that they should learn a ‘Lesson from the Seat of War’ and integrate Bovril into their defensive strategies against the enemy they face – influenza. The war against germs is thus as important as the war against the Boer republics, but immunity is depicted as the reward won by Bovril rather than the result of a process through which the body responds to infection. Within these advertisements immunity and the immunological conjure the state of being defended by Bovril rather than the defence of the state.

During the First World War Kruschen Salts, an aperient and diuretic tonic that claimed to have the ‘secret of immunity’ from rheumatism, lumbago, and sciatica, presented an advertisement that was almost parodic in its use of military language.

Your body is an armed camp, of which a host of microscopic corpuscles that travel with your blood stream are the natural defenders. So long as your blood is pure and virile these defending hosts have all they ask – a fair field – to resist the attacks of invading organisms that threaten from without.^65^

The scene of friend and foe is complicated when the copy explains that there are ‘other even more insidious foes – traitors *within* the camp’. Hiding within the body are ‘a horde of enemies – pus-germs – which generate a certain virus and insinuate it, if you give them half a chance, into your blood-stream’. But, if Kruschen Salts are used to flush waste products from the body, then the blood becomes purified and the ‘pus-germs’ cannot generate their poison. This advertisement uses military language to explain the immune system and goes further in a description of immunological processes than the Bovril advertisements, but once again, immunity is presented as the benefit of the judicial use of a consumer product. Rather than the ‘host of microscopic corpuscles’ being presented as part of the body’s immunological defences, ‘immunity’ is not mentioned until the end of the fight, implying that immunity is simply the name of a resistant state attained by purchasing the product, which purifies the blood and thereby enables the body to fight off infection.

A Kruschen Salts advertisement from the 1918–19 pandemic is particularly illustrative.^66^ The copy begins: ‘*arising* doubtless from various irregularities – dietetic, sanitary, occupational, etc. – due more or less directly to the War, a number of mysterious maladies have recently broken out’.^67^ This malady, the copy explains, was first thought to be a form of botulism, but was later ‘nick-named “*What*ulism” for want of definite identification’. Explaining that the epidemic is linked to influenza, and providing images of the Pfeiffer bacillus, staphylococcus, and streptococcus – at the time the Pfeiffer bacillus was thought to be the cause of influenza rather than a secondary bacterial infection – the copy continued to warn readers to beware, as ‘Before we know it Influenza or La Grippe may be upon us in deadly earnest, and ourselves under the domination of enemies more ruthless and destructive even than the Hun’. Explaining that nature’s defensive system involves red and white corpuscles, ‘the natural defenders of the central citadel – the nervous system – of your health’, it outlines their specific functions in war metaphors: ‘The former energise your body to resist infection generally; the latter raid these “Germ-Huns” in their trenches, kill and (whisper it!) devour them bodily’.

The advertisement’s description of erythrocytes and phagocytes is basically accurate and provides an excellent example of the use of battle language to describe immunological processes. Significantly, however, there is no reason why the casual reader would have understood this process to be immunological, as immunity is positioned as the resistance achieved by giving the ‘corpuscles *a fair field* whilst they fight your battles for you’, that is, by taking Kruschen Salts regularly. The nervous system is mentioned, and the advertisement claims that once ‘your blood be pure and virile there is not a germ in all bacteriology that has the power to impair your energy, efficiency and general well-being’, but ‘the secret of immunity from epidemics’ is presented as the final goal of a Kruschen habit, that is, of taking Kruschen salts regularly. That immunity stems from the process involving the red and white blood cells that the advertisement mapped out is de-emphasised. Once again, the shielding power of immunity is evoked, only for the power to be shifted from immunological processes, and what we would now call the immune system, to the product. The danger of this depiction is that it externalises immunity and entrenches it in a purchasable commodity, thereby potentially replacing, in the public’s mind, cellular processes with capitalist processes. If immunity is the pinnacle of health, then health, within this logic, is innately something you buy and achievable only to those with open wallets, which leads us to the associations forged between immunity and futurity, as well as immunity and communities.

## Immunity to come

**Figure 5.**
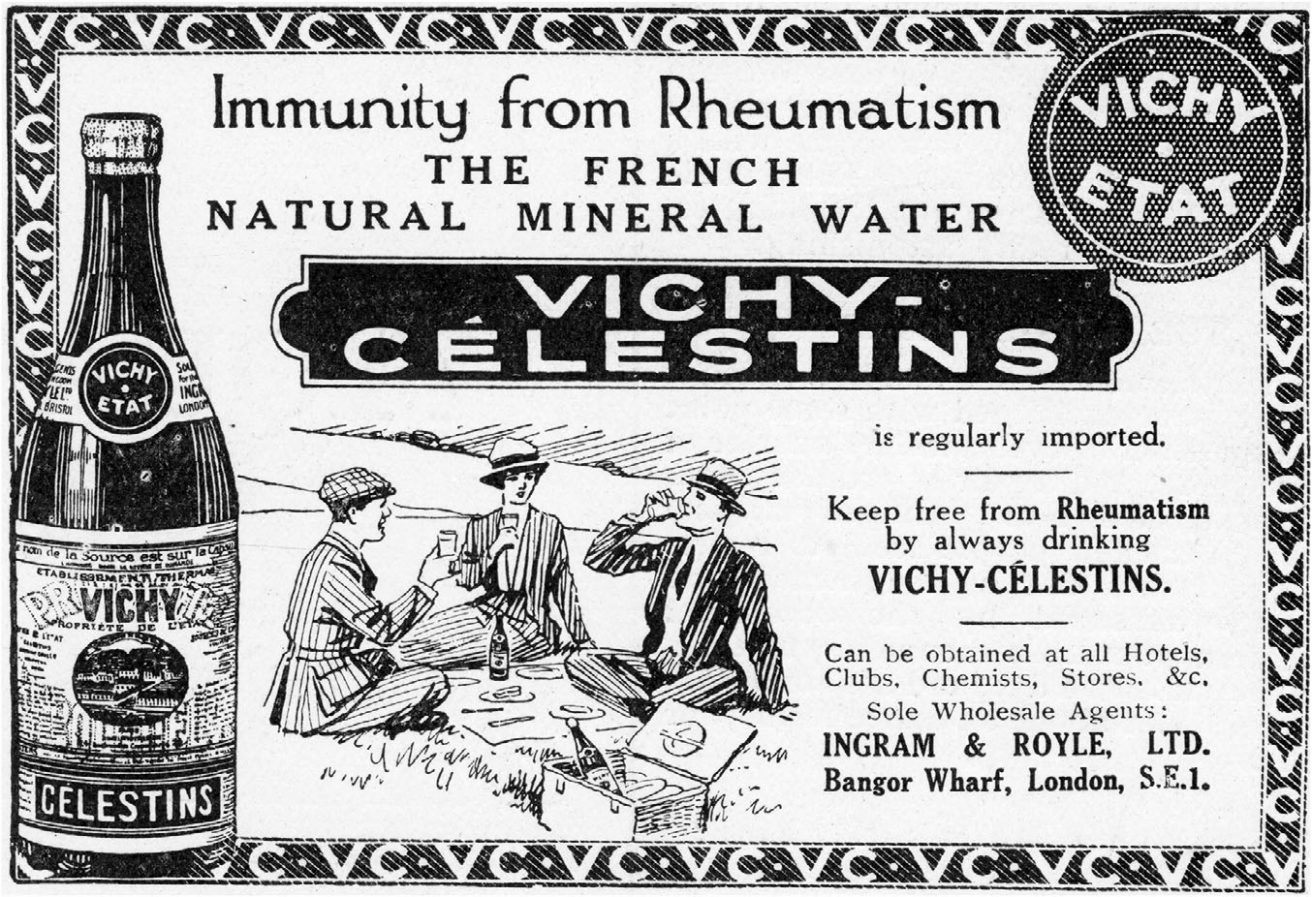
Vichy Célestins. Source: *The Graphic*, 4 June 1921, 2. © Illustrated London News Ltd/Mary Evans.

Many advertisements by dentists or of dental products promised immunity from pain, such as the wonderfully named Yu-Suth-Me, which claimed to give ‘longer immunity from pain than any other medical preparations’.^68^ However, the majority of advertisements that drew on immunity, particularly as the twentieth century advanced, presented their products as preventative measures that enhanced natural immunity: tonics or pills that purified the blood or increased vitality and thereby enabled the body to resist disease. For most of the 1920s Vichy-Célestins mineral water was sold under the tagline ‘Immunity from Rheumatism’ (Figure 5), with the promise that regular consumption would keep the consumer rheumatism-free.^69^ Even food was caught up in ideas of preventative medicine: Atora Hugon’s Beef Suet, for example, was specifically sold as one of the ‘“*protective foods*”’ that gives ‘immunity from Rickets and Tuberculosis’.^70^

In the advertisements published in the early twentieth century, prevention and potential become evocatively entangled via immunity’s futurity. When Holloway’s Pills promised ‘immunity from the dangers and pangs of disease’^71^ they were selling the concept of a preventative against future threats, a shield erected in the present for threats to come. We can add nuance to this claim by considering viruses dormant within the body and asymptomatic carriers, but in terms of the definitions available to the public in dictionaries and popular medical texts in the early twentieth century, you cannot be immune to the illness you currently have, only to the one yet to arrive. In this way, immunity is always future-focused. Immunity functions as an insurance policy that comes with absolute risk protection, protection of such iron-clad variety that it prevents the threat from materialising. But while to be immune is to be shielded from the germs breathed out by a co-worker or waiting on the handle of a door, some companies made the future focus of immunity and preventative medicine explicit. Bishop’s Lithia Varalettes, a cure for gout and rheumatism, are a fine example, as for decades they emphasised their products’ ability to immunise the user against future ills: ‘secure immunity from future suffering by the aid of Bishop’s Varalette’s’,^72^ they urged, as it is ‘the only remedy’ that ‘ensures immunity from future suffering’.^73^ Take a tablet now for ‘complete relief’ and with it you are assured of ‘future immunity from pain’.^74^

The temporal focus of the Bishop’s Lithia Varalettes advertisements was made spatial in advertisements for Simpson’s Iodine Locket from the late 1930s. These advertisements told consumers that the ‘germ-destroying power of *reinforced iodine* vapour’ was so strong that users could ‘live in a permanent “immunity zone”’.^75^ In this instance not only does risk become a thing of the past, enabling the discerning consumer to put fears of infection and suffering to one side forever, but their immunity is so strong that it encases them in a place of exclusion and separation. Their immunity is territorialised, marked by clear limits that cannot be traversed. The ‘immunity zone’ that the advertisement describes is not simply for the individual, however, as ‘thousands of other people have for years’ lived there, simply by wearing their iodine lockets. The immunity zone becomes a community zone, a utopian space in which ills have been eradicated. Yet, as this immune community depends on strict and fixed borders, with threats positioned on the outside of the zone’s walls, we can see clearly how immunity could be readily co-opted by nationalist and xenophobic discourses. If the immunity zone is populated by individuals who wear the same iodine-diffusing locket, outside the zone lie the people who do not: the people who are not immune, the people dwelling amongst the illness, the people whose future holds no hope. Those, to return us to the impact of the externalisation of immunity, who lie outside the promises of a commodified, capitalist immunity.

These people are, other advertisements indicate, different from those with immunity because they lack the purity of blood inherent to the state of being immune. In the *Eugenics Review* in 1912, J.A. Lindsay, chair of medicine at Queen’s University Belfast, argued that even if ‘conditions which confer immunity upon individuals and upon races’ were difficult to predict, questions of immunity to disease were some of the ‘most profound problems of genetics, of eugenics, of racial fitness and survival and of racial unfitness and decay’.^76^ The traces of such ideologies can be found across the advertising copy of the period, particularly in tonics, with an advertisement for Roboleine tonic in 1925 making the eugenicist leanings of blood discourses particularly overt: ‘Make them [children] *fit* to take their pleasures *gladly*’. In this advertisement the ‘immunity from illness’ Roboleine gives is the hallmark of the vital body, less visible than ‘rosy cheeks’ but an equally necessary sign of ‘good *blood*’.^77^ Although the overt eugenicist leanings decreased over time, the impacts of this rhetoric continued into the 1930s, with Sanatogen tonic, for example, connecting immunity, blood, and purity by regularly warning of the dangers of ‘weak blood’ in 1935.^78^ The immunity zone could readily lend itself to the rhetoric of eugenics and discrimination, marked by a boundary between the healthy and the unhealthy, the fit and unfit, the pure and the impure. Recognising these early biopolitical uses of immunity gives us an important context from which to understand work done on immunity, community, and biopower in the late twentieth and early twenty-first centuries by Roberto Esposito, Peter Sloterdijk, Ed Cohen, and Nik Brown.^79^

## Immunity against worry

Finally, we turn to the ways narratives of immunity intersected with wider concerns about anxiety and security. As outlined earlier, Brown’s *Worry, and How to Avoid It* posited that mental exercises would give individuals immunity from worry. Copywriters for Beecham’s Pills, who were also aware of the prevalence of uncertainty and apprehension in the early twentieth century, argued that their products would render users even more immediately and easily immune. Beecham’s Pills, which were notorious for their large advertising budget, were consistent users of immunity in their advertising between the 1900s and the mid-1920s. These advertisements, which ran for extended periods over numerous newspapers, and promised cures to digestive issues, pain, disease, tired countenances, colds, and rheumatic pain, were lifestyle advertisements that connected immunity to happiness, good luck, and well-being, specifically presenting Beecham’s Pills as supplements that eliminated worry and nervous collapse along with ill-health. As such they drew on wider arguments about the importance of ‘welding the physical and the psychical into a science of health’^80^ and, like Brown, implied that one could be immunised against anxiety.

**Figure 6.**
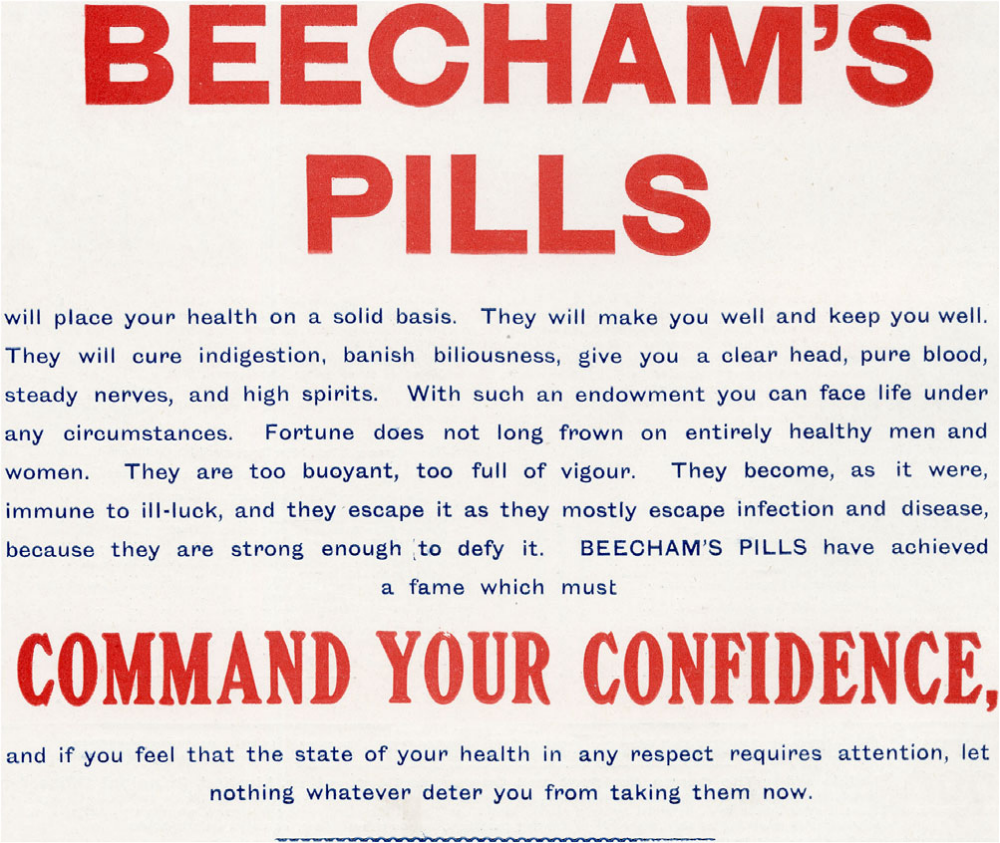
Section of Beecham’s Pills advertisement. Source: *The Tatler*, 9 October 1907, 40. © Illustrated London News Ltd/Mary Evans.

In 1906 an advertisement campaign declared that the majority of those ‘who enjoy perfect health […] owe their immunity from illness to *Beecham’s pills*’.^81^ Those who are healthy, the advertisement explained, are the ‘happy ones’ who are free from ‘depressing sensations’ and elevated by the ‘delightful consciousness that all is well’. The Beecham’s user was a healthy, carefree individual, unworried by the challenges of life, and as further advertisements argued, rendered immune from misfortune. Although ‘Sickly, anaemic, dyspeptic, nervous people can never be happy’, the copy declaimed,

Fortune does not long frown on entirely healthy men and women. They are too buoyant, too full of vigour. They become, as it were, *immune to ill-luck*, and they escape it as they mostly escape infection and disease, because they are strong enough to defy it’.^82^ (Figure 6)

If, as Brown writes, ‘worry is supposed to be incurable because medicine does not satisfactorily minister unto it’,^83^ Beecham’s Pills begged to differ. Their advertisements claimed to enable users to be free of concerns about health. Healthy, they were worry-free, and worry-free, they would be even less likely to ever fall ill. According to Beecham’s, immunity to illness meant immunity to the vicissitudes of fate.

Risks were dealt with directly in a series of advertisements, printed frequently during the First World War, that connected immunity with generalised security. ‘Security’, these Beecham’s advertisements insisted, ‘is a comprehensive word indicating a position which everybody wishes to stand in. The desire for security is indeed one of the most natural cravings to be found in any normal person. Who does not desire security in such matters as business, possessions, health?’. In the case of health, the copy claimed, ‘great immunity from illness is enjoyed by those who know how to preserve their digestive system’.^84^ By using an almost identical pattern to the cheerfulness advertisements, immunity, security, protection from bad luck, and cheerfulness are entangled, with each instance evoking the other terms. Even though the copy does not mention the war, and specifically highlights the ways a well-functioning digestive system counters the worries and strains of modern life, the evocation of security – comprehensive security – during a world war would have been particularly powerful. In as much as William S. Sandler saw the ‘science of self-mastery’ as the means to defeat worry, Beecham’s Pills presented their products as part of an arsenal of self-treatment, vital ingredients in enabling immunity against the weaknesses of body and mind.^85^

## Conclusion

The early twentieth century saw a marked growth in the concept of immunity as a sales pitch, with complete and permanent resistance to a wide manner of illnesses regularly promised by a broad variety of patent medicines. Over the decades the style of advertising changed, as lengthy pitches were gradually replaced by pithier copy and a greater use of images. Immunity as a tool in the advertiser’s cache grew from rare and sporadic mentions in the 1890s, as immunity was still making its way from science laboratories to social awareness, to peak in the 1920s when it had sustained use across products and advertising campaigns, promising immunity to a population striving for security and balance after a world war and an influenza pandemic. It featured slightly less frequently in the 1930s and into the 1940s, but this decrease in quantity was tempered by greater advertiser confidence in the public response to the term, a confidence that manifested in their increased use of immunity in taglines, as the major pitch in succinct copy, and in bolded text.

Collectively, the immunity presented by the advertisements published in British newspapers between 1890 and 1940 was deeply embedded in a magical thinking about health: health that could be inviolate and impregnable if the right price was paid. Purchasing patent medicines became, according to the language of advertising, acts of painless, needle-free vaccination. Every glass of a tonic or mineral water, every suck on a lozenge, every swiftly swallowed pill combined, marketing insisted, to create a body with impenetrable boundaries against threat, from the annoyances of discomfort to the risks of death. As the sections above show, immunity was often decoupled from immunological processes and instead presented as a state deeply embedded in capitalist, transactional exchange and achieved by the prudent use of proprietary medicines. Immunity was something you bought, which welded it to products and purchasing power, even when it was strategically embedded in the lure of ‘natural’ interventions and natural immunity. The rhetoric of nature was frequently aligned to discourses of healthy blood and fit bodies – with both health and fitness inextricable from purchasing power – which brought immunity into the realms of eugenicist divisions and social exclusion. And, as we have seen, this nexus of social hierarchies, capitalism, and nature was extended to include growing anxieties about mental health, such that the discourse of a purchasable community promised a life absolutely protected from worry and concern.

Advertising language, particularly the hyperbolic address of patent medicine marketing, is not an everyday language, but the advertising agent can be considered, as the revolutionary English journalist William Stead wrote in 1899, the

nerve-centre of modern industry. He is the first to feel every influence which affects industry, whether for good or ill. He keeps, as it were, his finger upon the commercial pulse of the world, counts his beats, and adjusts the method and quantity of advertising accordingly’.^86^

Even if the presence of immunity in advertising copy does not give us unfettered access to the way the public used immunity, it affords us significant insights into the ways the newspaper-reading public was exposed to the concept of being medically immune as well as the range of meanings immunity encapsulated. By examining the use of a rhetoric of immunity in advertising copy during the late nineteenth and early twentieth centuries, a period that marks the beginning of modern immunology, we gain new insights into the ways immunity was presented to the public outside of medical pamphlets and doctors’ offices. These insights – the frequent decoupling of the term from immunological processes to emphasise magical thinking about immune shields and immune lives, and the term’s malleability across ideological positions – give us a new historical base from which to view the discourses of immunity prevalent in late twentieth and early twenty-first-century public health crises, from AIDS to COVID-19. As Davis notes in his recent analysis of immunity in the rhetoric of contemporary healthcare, the depictions of immunity that ‘took shape in the late nineteenth century are not overturned and displaced’ in discourses today, but ‘persist and are modified as they are brought into connection with new knowledge, styles of science, and economic rationality’.^87^

## Footnotes

1 With thanks to Jessie Burnette, Pascale Grard, Ross Lawrenson, and Marie Theunisz.

2
*Globe*, 31 August 1899, 6.

3
*Nottingham Evening Post*, 11 February 1909, 7.

4
*Dundee Evening Telegraph*, 4 May 1909, 6. For scepticism on their right to such claims see *Report from the Select Committee on Patent Medicines, together with Proceedings of the Committee, Minutes of Evidence, and Appendices, Ordered by the House of Commons, to be Printed* (London: Wyman and Sons, Ltd., 1914), xvii.

5
*The Tatler*, 11 July 1923, 45.

6
*Daily News*, 31 December 1938, 16.

7 The idea of an immune *system* dates from the 1960s. See, for example, David Napier , ‘Nonself Help: How Immunology Might Reframe the Enlightenment’, Cultural Anthropology, 27, 1 (2012), 122.22451984 10.1111/j.1548-1360.2012.01130.x

8

H. G. Wells , The War of the Worlds (London: William Heinemann, 1955), 210.

9 James Anderson chemist, *Dundee Evening Telegraph*, 21 November 1933, 6. See too Martina King , ‘Anarchist and Aphrodite: On the Literary History of Germs’, in Thomas Rütten and Martina King (eds), Contagionism and Contagious Diseases: Medicine and Literature 1880–1933 (Berlin, 2013), 101–30.

10

G. B. Shaw , The Doctor’s Dilemma, Getting Married, & The Shewing-up of Blanco Posnet (London: Constable and Company, 1927), xxxiii.

11
*Lloyd’s Weekly Newspaper*, 20 February 1910, 23.

12 Lifebuoy Soap, *Derby Daily Telegraph*, 23 April 1914, 4.

13

Arthur M. Silverstein , A History of Immunology (San Diego: Academic Press, 1989); Kenton Kroker , Jennifer Keelan and Pauline M. H. Mazumdar , eds, Crafting Immunity: Working Histories of Clinical Immunology (London: Routledge, 2016).

14 Ilya Mechnikov (Élie Metchnikoff), ‘On the Present State of the Question of Immunity in Infectious Diseases’, Nobel lecture, 11 December 1908. https://www.nobelprize.org/prizes/medicine/1908/mechnikov/lecture/

15 See, for example, Allen J. Smith’s *A Practical Text-Book of Infection, Immunity and Biologic Therapy* (Philadelphia and London: W. B. Saunders, 1925), where immunity is defined ‘not only the ability to resist an infection or successful invasion of the tissues by microparasites, but also the continued resistance offered as long as the infection lasts’ (199).

16
*The Modern Family Doctor: A Guide to Perfect Health* (London: T.C. and E.C. Jack, 1914), ix.

17 See, for example, Heather A. Love and Lisa Mendelman’s cluster on ‘Modernism and Diagnosis’ in Modernism/modernity PrintPlus 6, 2 (2021), np., https://doi.org/10.26597/mod.0198 and Beth Blum’s *The Self-Help Compulsion: Searching for Advice in Modern Literature* (New York: Columbia University Press, 2020). Samuel Smile’s *Self-Help, with Illustrations of Conduct and Perseverance* (Chicago: Belford, Clarke & Co., 1859) is considered to be one of the first self-help books.

18 Lori Loeb, ‘Doctors and Patent Medicines in Modern Britain: Professionalism and Consumerism’, *Albion: A Quarterly Journal Concerned with British Studies* (2001), 404-25.

19 Francis O’Gorman, ‘Modernism, T.S. Eliot, and the ‘age of worry”, *Textual Practice*, 26, 6 (2012), 1001–19; Lauren Alex O’Hagan , ‘Blinded by science? Constructing Truth and Authority in Early Twentieth-century Virol Advertisements’, History of Retailing and Consumption, 7, 2 (2021), 162–92.

20

Andrew Wilson , Brain and Body: The Nervous System in Social Life (London: James Bowden, 1900), 10.

21
*Ibid.*, 12.

22

C.W. Saleeby , Worry: The Disease of the Age (London: Cassell and Company, 1907), 29–30.

23 Haydn Brown, *Worry, and How to Avoid It* (London: James Bowden, 1900), 13.

24
*Ibid.*, 37.

25

M. Beddow Bayly , Natural Immunity: A Lecture (London: The Health Education and Research Council, 1938), 24 . The lecture was given under the auspices of the Health Education and Research Council in London in 1937.

26

T.A.B. Corley , *Beecham’s 1848*–*2000: From Pills to Pharmaceuticals* (Lancaster: Crucible Books, 2011), 2; Anat Rosenberg , The Rise of Mass Advertising: Law, Enchantment, and the Cultural Boundaries of British Modernity (Oxford: Oxford University Press, 2022), 195.

27
*Chemist and Druggist*, 26 June 1886, 630. Qtd. Corley, *Beecham’s 1848-2000*, 22–3.

28 British Medical Association, *Secret Remedies: What they Cost and what they Contain* (London: British Medical Association, 1909), 9.

29

Takahiro Ueyama , Health in the Marketplace: Professionalism, Therapeutic Desires, and Medical Commodification in Late-Victorian London (California: The Society for the Promotion of Science and Scholarship, 2010), 84–5.

30 See, for example, Nadja Durbach , Bodily Matters: The Anti-Vaccination Movement in England 1853–1907 (Durham, NC: Duke University Press, 2005), 167.

31

Patrick Berche , ‘The Enigma of the 1889 Russian Flu Pandemic: A Coronavirus?’, Presse Medicale, 51, 3 (2022), 104–11.10.1016/j.lpm.2022.104111PMC881372335124103

32

Lori Loeb , ‘Beating the Flu: Orthodox and Commercial Responses to Influenza in Britain, 1889–1919’, Social History of Medicine, 18, 2 (2005), 203–24.

33
*Ibid.*, 213.

34

Raymond Williams , ‘Advertising: The Magic System’, in Simon During (ed.), The Cultural Studies Reader (London: Routledge, 1993), 410–26, 422 .

35
*Ibid.*, 422.

36
*Gloucester Citizen*, 10 December 1931, 5.

37
*Hull Daily Mail*, 1 March 1932, 3. Emphasis in original.

38

T. J. Jackson Lears , ‘From Salvation To Self-Realization: Advertising and the Therapeutic Roots of the Consumer Culture, 1880–1930’, Advertising & Society Review, 1, 1 (2000), np.

39

Mark Davis , Selling Immunity: Self, Culture and Economy in Healthcare and Medicine (London: Routledge, 2022), 81.

40
*Nottingham Evening Post*, 23 August 1918, 4. Emphasis added.

41
*Hull Daily Mail*, 12 January 1922, 3. Emphasis added.

42
*Daily News*, 2 June 1928, 6. Emphasis in original.

43
*Ibid.*, 17 October 1916, 6.

44
*Cheltenham Chronicle*, 3 September 1938, 5. Emphasis in original.

45
*Yorkshire Evening Post*, 17 April 1902, 3. Emphasis added.

46
*Empire News and the Umpire*, 1 March 1908, 12.

47
*Aberdeen Press and Journal*, 31 May 1938, 9. Emphasis in original.

48
*The Tatler*, 30 November 1927, 101.

49
*The Tatler*, 24 February 1926, 67.

50
*Belfast News-Letter*, 14 March 1934, 10.

51 Walter R. Hadwen, ‘An Unnatural Practice’, *The Abolitionist: The Journal of The British Union for the Abolition of Vivisection* (1921), 13–14, 14.

52
*Yorkshire Post and Leeds Intelligencer*, 19 March 1936, 1. Emphasis added.

53
*Daily News*, 20 January 1937, 7.

54
*Ibid.*, 9 February 1937, 7. Emphasis added.

55

Emily Martin , Flexible Bodies: Tracking Immunity in American Culture from the Days of Polio to the Days of AIDS (Boston: Beacon Press, 1994); Fred Karush , ‘Metaphors in Immunology’, in Pauline M. H. Mazumdar (ed.), Immunology 1930–1980: Essays on the History of Immunology (Toronto: Wall and Thompson, 1989), 73–80 ; Mark Davis , Selling Immunity: Self, Culture and Economy in Healthcare and Medicine (London: Routledge, 2022).

56
*Western Daily Press*, 11 January 1902, 3.

57
*North West Evening Mail*, 25 January 1911, 3. Emphasis in original.

58
*Daily News*, 17 October 1916, 6.

59

Susan Sontag , Illness as Metaphor and AIDS and its Metaphors (London: Macmillan, 2001); Emily Martin , ‘Toward an Anthropology of Immunology: The Body as Nation State’, Medical Anthropology Quarterly, 4, 4 (1990), 410–26.

60

Elizabeth T. Fraser , A Manual of Immunity for Students and Practitioners (Glasgow: James Maclehose and Sons, 1912), 70.

61 See, for example, Donald J. Childs , Modernism and Eugenics: Woolf, Eliot, Yeats and the Culture of Degeneration (Cambridge: Cambridge University Press, 2001).

62

Angela Loxham , ‘Profiting from War: Bovril Advertising during World War II’, Journal of Macromarketing, 36, 2 (2016), 198–214, 200.

63
*Daily News*, 16 December 1899, 1. Emphasis in original.

64
*Evening News*, 14 December 1899, 4.

65
*The Sphere*, 10 March 1917, 44.

66 For another reading of medical advertising in Britain during the 1918–19 pandemic, see Lauren Alex O’ Hagan , ‘Commercialising Public Health during the 1918–1919 Spanish Flu Pandemic in Britain’, Journal of Historical Research in Marketing, 13, 3/4 (2021), 161–87.

67
*The Graphic*, 6 July 1918, 21. Emphasis in original.

68
*Southern Reporter*, 16 November 1905, 4.

69
*The Graphic*, 4 June 1921, 2.

70
*Western Evening Herald*, 11 January 1923, 4. Emphasis in original.

71
*Western Times*, 12 March 1900, 2.

72
*The Graphic*, 6 July 1907, 30.

73
*The Graphic*, 23 May 1908, 22.

74
*Western Morning News*, 25 September 1923, 8.

75
*Dundee Courier*, 13 December 1939, 6. Emphasis in original.

76

J.A. Lindsay , ‘Immunity from Disease Considered in Relation to Eugenics’, The Eugenics Review, 4, 2 (1912), 117–35, 13521259532 PMC2986833. For more on immunity and race, see Warwick Anderson, ‘Immunities of Empire: Race, Disease, and the New Tropical Medicine, 1900-1920’, *Bulletin of the History of Medicine*, 70, 1 (1996), 94–118.

77
*Daily News*, 22 May 1925, 2. Emphasis in original.

78 See, for example, *Dundee Courier*, 28 June 1938, 5.

79

Roberto Esposito , Immunitas: The Protection and Negation of Life (Cambridge: Polity, 2011); Peter Sloterdijk, *Bubbles: Microspherology* (Los Angeles: Semiotext(e), 2011); Nik Brown , Immunitary Life: A Biopolitics of Immunity (London: Palgrave Macmillan, 2019); Ed Cohen , A Body Worth Defending: Immunity, Biopolitics, and the Apotheosis of the Modern Body (Durham: Duke University Press, 2009).

80 C.W. Johnson, *Health: Physical and Mental or Hygiene of Body and Mind with Hints on Prevention and Cure of Disease Through Cultivation of Natural Immunity* (London: The Theosophical Publishing Society, 1909), 16.

81
*Bridlington Free Press*, 7 December 1906, 2. Emphasis in original.

82
*The Tatler*, 9 October 1907, 40. Emphasis added.

83 Brown, *Worry*, 17.

84
*Derby Daily Telegraph*, 23 June 1915, 4.

85

William S. Sandler , Worry and Nervousness or The Science of Self-Mastery (London: C. F. Cazenove, 1914).

86

William Stead, Jr., The Art of Advertising: Its Theory and Practice Fully Described (London: T. B. Browne, Ltd., 1899), 42.

87 Davis, *Selling Immunity*, 38.

